# Nutrient Composition, Sensory Qualities, and Acceptability of Locally Prepared Ready‐To‐Use Therapeutic Food in Northern Ghana's Hospital Settings

**DOI:** 10.1002/fsn3.70033

**Published:** 2025-02-10

**Authors:** Tamimu Yakubu, Charles Apprey, Reginald Adjetey Annan

**Affiliations:** ^1^ Department of Biochemistry and Biotechnology Kwame Nkrumah University of Science and Technology Kumasi Ghana; ^2^ Department of Nutrition and Dietetics Tamale Technical University Tamale Ghana

**Keywords:** acceptability, local therapeutic formula, nutrient composition, RUTF, SAM

## Abstract

Malnutrition is a significant life threat to children under 5 years of age, especially in disadvantaged regions where accesses to commercial therapeutic foods are limited. Local therapeutic foods, specifically Ready‐to‐Use Therapeutic Food (RUTF), have emerged as crucial interventions. This study delves into the nutrient composition, sensory qualities, and acceptability of hospital‐based locally formulated RUTF in comparison to the WHO standard formulation, examining samples from two hospitals in the northern region of Ghana. The research was a cross‐sectional study design, conducted within 3 months and involved 112 mothers/caregivers and their children. The locally prepared RUTF, demonstrated potential to meet the nutrient requirement of children under 5 years, particularly for SAM management. Microbial analysis indicated safe consumption levels, but discrepancies in nutrient composition arose due to ingredient variations and addition of specific minerals and vitamin. The local formulae compared to WHO standard RUTF, liquid therapeutic formulae, and human breast milk revealed both strengths and limitations in the local formulations. Chemical analysis of samples revealed energy and protein content of 530 kcal and 14 g per 100 g for Tamale Teaching Hospital (TTH), and King Medical Centre (KMC) recorded 570 kcal and 11 g per 100 g respectively. The mean and average acceptability of the samples indicate WHO standard RUTF performed better than the other two samples with an overall acceptability of (30.4) followed by TTH (27.0) and KMC was least liked with (23.0). The study highlights the need for stringent adherence to guidelines and local adaptations to ensure effective, culturally appropriate, and safe therapeutic formulae for malnourished children, and hence, emphasized the critical role of local solutions in global malnutrition management strategies.

## Graphical abstract

In two hospital settings in Northern Ghana, this study assesses the nutritional makeup, sensory attributes, and acceptance of locally prepared Ready‐to‐Use Therapeutic Food (RUTF). The results reveal that although locally produced RUTF has potential for addressing children's nutritional needs in the management of severe acute malnutrition (SAM), nutrient levels are impacted by ingredient variances when compared to the WHO standard. The WHO standard RUTF was preferred by acceptability testing, emphasising how crucial it is to match local adaptations with international standards in order to maximise safety and efficacy.

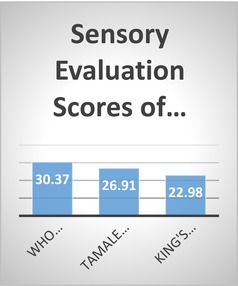

## 1 Introduction

Local therapeutic food plays a fundamental role in the management of malnutrition, particularly in disadvantage settings and regions where access to therapeutic commercial formulations are limited (Bailey et al. 2018; Kane et al. 2015). Malnutrition is reported to be attributable to over half of all mortalities in children under 5 years, and severe acute malnutrition (SAM) leads to one million of those mortalities annually (Black et al. 2013). The United Nations children fund (UNICEF) also reported that a child dies every 15 s due to malnutrition and infection, and that while undernutrition in the developed countries is dipping, the cases of undernutrition continue to rise in third world countries (Unicef 2013).

Therapeutic formulae, refers to foods that are nutritionally enhanced to have high energy and nutrients to attain the requirements of the severely malnourished child (Latham et al. 2011), and usually administered for therapeutic purposes as a form of dietary supplement. Locally prepared therapeutic foods have the potential to address nutrient needs by leveraging locally available ingredients. However, as far as the World Health Organization (WHO) protocol (WHO 2013; WHO and UNICEF 2007) of local formulation of Ready‐to‐Use Therapeutic Food (RUTF) is concerned in terms of its nutrient composition, sensory qualities, and acceptability compared to standard formulations have not been extensively studied (Eloho et al. 2017; Nga et al. 2013). Locally formulated RUTF has shown promising outcomes in clinical settings, including significant improvements in growth rates, weight gain, and overall health status among children suffering from malnutrition. These outcomes highlight the potential of locally formulated RUTF to effectively meet the nutritional needs of vulnerable populations (Thapa et al. 2017). Weber et al. (2017) reported that, standard RUTF is often labeled and stigmatized as an imported formula made with exogenous ingredients, and this behavior largely goes to impede smooth usage and widespread adoption.

Standard RUTF and Ready‐to‐Use Supplementary Foods (RUSFs), have been manufactured and implemented by transnational establishments to combat not only SAM, but malnutrition as a whole (LaGrone et al. 2012; Sigh et al. 2018). The RUTF is specifically designed for children diagnosed with SAM and is administered under medical supervision, while RUSF is intended for children at risk of malnutrition or those with Moderate Acute Malnutrition (MAM). These formulations usually comprise of nutrients and recipe that are high in energy, involving milk powder, oil, sugar, and fortified micronutrients (e.g., potassium, iron, magnesium, copper etc), which are processed into paste with little or no moisture, to prevent spoilage (Borg et al. 2019; Sigh et al. 2018). RUTF formulations in addition to providing high energy and nutrient density, also provides approximately 500 kcal per 92 g, with approximately 10%–12% of the total energy derived from protein, alongside essential fats and a range of micronutrients necessary for recovery and growth (UNICEF 2023). A number of studies have demonstrated the efficacy of standard RUTF in promoting weight gain, recovery, and improved health outcomes among malnourished individuals (Bahwere et al. 2016, 2014; Potani et al. 2021).

In many of the health settings in recent times, where SAM cases are managed at the inpatient level, there is difficulty in accessing the imported standard RUTF, and hence, health staff resorts to the local formulations of the RUTF. Also, Kohlmann et al. (2019) and colleagues reported from their research conducted in Ghana that, only 15% of SAM children receive RUTF globally (Kohlmann et al. 2019). The support of therapeutic formulae by UNICEF, has in recent times hugely constrained due to Covid‐19 pandemic and armed conflicts, including the war in Ukraine, driving up the prices of therapeutic formulas (UNICEF 2022), thus resulting in persistent shortage of these supplies affecting management of hospitalized case. This practise of managing cases using the local formulation has been on‐going for the past 5 years in the northern sector of Ghana, and shows promising outcomes in terms of weight gains, improvement in mid‐upper arm circumference (MUAC) and overall recovery. Thus, understanding the nutrient composition of locally prepared therapeutic food (i.e., RUTF) is essential as variations in nutrient content can significantly impact its effectiveness in the management of SAM (Schoonees et al. 2013). Additionally, sensory qualities and acceptability of these formulae play a vital role in ensuring compliance and adherence to the prescribed therapeutic formula regimen.

Consequently, if local formulations exhibit comparable functions to standard formulations, it could potentially provide a more sustainable and culturally appropriate solution for addressing malnutrition (Marchini et al. 2022). By appreciating the possibility of locally therapeutic formulation in making positive difference in the lives of the SAM children, this study can help improve these local formulations to enhance their efficiency and usefulness in the fight against malnutrition and SAM cases in general. The outcomes thereof will provide comprehensive insights for healthcare professionals, policymakers, and organizations involved in the general welfare of children with malnutrition. Therefore, this study sought to contribute to the area of SAM management by evaluating the nutrient composition, sensory qualities, and acceptability of RUTF prepared locally from Tamale Teaching Hospital (TTH) and Kings Medical Centre (KMC) compared to the standard formulation.

## 2 Materials and Methods

### 2.1 Study Design, Setting, and Duration

This study employed a cross‐sectional design that was conducted in the pediatric wards and child welfare clinics (CWC) at both the TTH and KMC in the Northern Region of Ghana. The study was carried out for 3‐months period to allow for an adequate sample size and data collection.

### 2.2 Participants

The study involved lactating mothers/caregivers with children between 6 and 59 months at the CWC and pediatric wards of TTH and KMC. Thus, it involved children aged 6 to 59 months, who were assessed for their acceptability of the therapeutic food samples. The study excluded mothers of children with other medical conditions i.e. those with peanuts allergy or those receiving other forms of nutritional intervention. The sensory results were obtained from the mothers or caregivers as the case may be, who provided feedback on their children's preferences and reactions to the samples under consideration. This distinction is important for understanding the context of the acceptability ratings, as the caregivers' insights reflect the children's experiences during the tasting sessions.

### 2.3 Sample Size

The study by Hough et al. (2006), employed 112 respondents necessary for sensory acceptability tests in food quality and preference, using 5% type 1 error, 10% type 2 error and sample mean of 10% of the sensory scale (Hough et al. 2006). Therefore, in line with Hough and colleagues; this current study recruited 112 mothers/caregivers who had children between 6 months and 59 months. Purposive sampling technique was used to get eligible mothers or caregivers for this procedure.

### 2.4 Formulation Procedure for Local RUTF

The therapeutic foods were prepared at diet kitchen or nutrition laboratory at TTH and KMC. All the ingredients were bought at the Tamale Central Market and Kumbungu District Market for the preparation of the therapeutic formulae, for TTH and KMC respectively. As far as the ingredients were concerned, both hospitals used similar ingredients for the local formulation, except skimmed milk powder, where TTH used “Nido Essentia” brand and KMC “Lactogen 2”, all products of Nestle‐Ghana. The proportions of the ingredients were based on the total weight of the therapeutic food as follows; peanut butter or groundnuts = 25%; milk powder (skimmed & whey powder) = 30%; vegetable oil = 15%; sugar = 28%; and mineral mix (Zincovit) = 1.6%. The ingredients were weighed and measured accurately according to the desired proportion required. The groundnuts were roasted using local dry skillet until they turn golden brown and became fragrant. The groundnuts were allowed to cool. The roasted groundnuts were milled and ground into a smooth paste. In a mixing bowl, the groundnut paste (or peanut butter), milk powder, vegetable oil, sugar, and the desired quantity of the Zincovit was mix thoroughly. Ingredients were mixed well to ensure it was evenly distributed. Mixture was transferred back to the blender and blended for 5 min until a homogenous and smooth consistency was achieved. The blending process helped in ensuring proper mixing of ingredients and obtaining a paste‐like texture. Mixture was scooped into a clean, airtight container, and was sealed tightly. The formulated RUTF products were stored under controlled conditions with a recommended shelf life of 12 months when kept at a temperature of 20°C–25°C. It was stored in a cool and dry place away from direct sunlight. The container was labeled with the date of preparation on it. The local prepared formula is a ready‐to‐use therapeutic food which is usually used to manage SAM in children under 5 years of age. During this local preparation process, good hygiene was maintained.

### 2.5 WHO Standard RUTF

The WHO standard RUTF utilized in this study was sourced from UNICEF in the northern region of Ghana, through the Regional Health Directorate‐Nutrition Unit. This formulation is designed to meet the nutritional needs of children suffering from SAM and adheres to the guidelines set forth by the WHO. The WHO standard RUTF is composed of a blend of energy‐dense ingredients, including peanuts, sugar, vegetable oil, milk powder, and a combined mineral and vitamin mix (CMV). The typical nutritional composition per 100 g of the WHO standard RUTF includes; 500–550 kca, 10–12 g of proteins, 45–60 g of fats and carbohydrates make up 30–40 g. This formulation is designed to provide the necessary macronutrients and micronutrients to support recovery in children with SAM. The production date of the WHO standard RUTF used in this study was April 28, 2023, with batch number 23175002 and it had an expiry date of April 2025. All RUTF samples were stored under recommended conditions to maintain their quality and efficacy until the time of use. This ensures that the RUTF used in this study was within the recommended shelf life for optimal nutritional efficacy and safety.

### 2.6 Serving Pattern of Therapeutic Food to Participants

The three samples of therapeutic formulae (i.e., from TTH, KMC, and WHO standard RUTF) were coded with random three‐digit numbers each (i.e., 561, 654, 458) respectively. The samples were served to each child one after the order. Thus, the first sample served was removed before the second and then the third sample given. The food samples were tasted one after the other. Participants were made to rinse their mouth with clean water before serving the other samples. There was a time delay of 6 min between servings.

### 2.7 Procedure for Serving Therapeutic Formulae

The research evaluators explained the objective of the acceptability study to the mothers/caregivers. The mothers and their children were made to undergo the standard practice of hand washing; by washing their hands under running water. Further, they were given paper towels. The participants were served with 5 to 10 g of the assigned samples for acceptability. The food was served in a 4 oz. disposable plastic cups and spoons, which was identified by the three random code numbers. Participants evaluated all the three samples using same questions for all the samples.

### 2.8 Sensory Evaluation

The mothers or caregivers were asked to sign a consent form. The activity was conducted in a controlled and comfortable environment and no any distractions during the evaluation. A team of evaluators (i.e., nutrition officers) were trained and ensured they understood the 9‐point hedonic scale thoroughly. The sensory evaluation was conducted immediately after the local formulation of the RUTF to seven (7) days postpreparation. This approach allowed for an assessment of the formulation acceptability over time. A questionnaire was used to elicit data on the three therapeutic formulae. The questionnaire was designed in a tabular format, and the responses from the participants recorded based on answers to the various attributes of interest. Thus, the 9‐point hedonic scale was explained to caregivers to understand the ratings of the attributes.

Caregivers were made to observe and interact with their children during the tasting session. Evaluators recorded the response to the assigned formulation on the 9‐point hedonic scale. The evaluators also encouraged verbal feedback from caregivers regarding the child's preferences. Attention was shown to child's facial expressions, body language, verbal cues (if any), and overall demeanor during the testing process. Largely, because the sensory evaluation involved children (6–59 months), caregivers were instructed to observe their reactions during tasting, as direct feedback may be limited due to their developing motor skills. Hence, both the children's responses to the RUTF formulation and the mothers' observations were considered, as mothers also tasted the products to corroborate their children's reactions. This method aligns with practices in previous research, where caregiver observations were used to enhance data collection (Ciliberto et al. 2006). Evaluators rated the samples on the following set of attributes namely appearance, consistency, texture, sweetness, and flavor/taste using a standard 9‐point hedonic scale, in which 1 was equal to dislike extremely and 9 was equal to like very much. Thus, if the child showed strong signs of disliking the formulation, we assigned a low score such as 1, 2, or 3. Also, if the child showed a neutral or mixed reaction, we assigned a mid‐range score like 4, 5, or 6. Then, if the child showed strong signs of liking the formulation, we assigned a high score like 7, 8, or 9. In addition to assigning a numerical score, caregivers were encouraged to provide qualitative feedback about their child's response to corroborate the evaluators' scores. This included comments on taste, texture, smell, and any specific preferences or dislikes. It was imperative to remember that this approach was indirect and relies on evaluators' interpretation. While it could provide valuable insights into children's acceptance of the therapeutic food, it was used in conjunction with caregiver feedback and child behavior during feeding, to create a more comprehensive picture of acceptability in children under 5 years.

### 2.9 Sampling of RUTF for Nutritional and Microbial Analysis

A total of 4 batches of RUTF were produced at each facility. Each batch was prepared using standardized recipes that adhered to the guidelines stipulated by WHO for therapeutic foods. The RUTF samples for microbial and nutritional analysis, were collected from each batch. Samples were randomly selected from each batch to ensure representativeness, i.e. approximately 100 g of RUTF was collected from each batch. Samples were taken immediately after preparation and before distribution to ensure that the microbial and nutritional content reflected the freshly prepared product. The collected samples were stored in sterilized containers and transported under controlled conditions for analysis at the laboratory. All samples were analyzed within 48 h of collection to maintain their integrity. As far as the microbial analysis was concerned, it was conducted following established guidelines, which included testing for coliforms, yeast, and *Staphylococcus aureus* , as recommended by WHO (2007) (WHO and UNICEF 2007). The results were compared against safety standards to ensure the RUTF was safe for consumption. Nutritional analysis was employed to determine the proximate and minerals composition of the RUTF, including protein, fat, and carbohydrates. The minerals analyzed were, copper, iron, potassium, magnesium, calcium, and zinc.

### 2.10 Therapeutic Food Composition Analysis

The nutrient content of the therapeutic food was analyzed at the Food Science Laboratory at the Kwame Nkrumah University of Science and Technology (KNUST). The nutrients analyzed were macronutrients (protein, carbohydrates, and fats), minerals (potassium, magnesium, calcium, zinc, copper and iron), and energy density. The proximate analyses of the samples were determined by using the standard procedures of Association of Official Analytical Chemist. The oven drying method was used to determine the moisture content (18). The protein content was determined by Kjedahl method (AOAC 1990), and also the composition of fat was by the Rose‐Gottlieb method. Additionally, ash was analyzed by dry ashing method by placing the sample in a muffle furnace and ignited for 12 h at 550°C. Carbohydrate was determined by calculating the percent difference after the determination of the moisture; hence protein, fat, and ash were determined thereof. Total energy was measured by calculating the water factor. Potassium was measured by the flame photometry, whereas magnesium and calcium were determined by EDTA titration method. Lastly, minerals relating to iron, copper, and zinc were determined by the atomic absorption spectroscopy (AAS). Samples were analyzed in duplicates or triplicates to ensure accuracy and reproducibility.

### 2.11 Microbiological Analysis of Local Therapeutic Formula

#### 2.11.1 Enumeration of Aerobic Mesophiles (Viable Plate Count)

Approximately 0.1% sterile peptone water or Butterfield's Phosphate Buffer (BPW) solution was prepared. Test sample was weighed and diluted with sterile diluent. Further dilutions were prepared to obtain countable colonies on agar plates. The diluted sample was then transferred to sterile petri dish. A molten Plate Count Agar was added, mixed uniformly, and solidified. Overplay was added for enhanced growth. The steps were repeated for each dilution. Agar plates were incubated upside down at 32°C ± 1°C for 48 h. Colonies were counted on each plate using a colony counter. Colony counts were recorded, and calculated average count. We calculated aerobic plate counts (APC) using formula: APC = (average colony count × dilution factor)/Volume of inoculum used. APC was expressed as colony forming units (CFU) per gram. Positive and negative controls for accuracy were performed. The equipment was then sterilized, and made to follow good laboratory practices and safety guidelines. Scheme of analysis for enumeration of aerobic mesophilic bacteria in the local prepared therapeutic formulae was done using the plate count method (APHA 8:2015).

#### 2.11.2 Enumeration of Yeast and Molds (TYMC)

Sterile peptone water (0.1%) or BPW were prepared. The test sample were prepared and diluted with sterile diluent. Also, further dilutions were created for countable colonies on agar plates. Then the diluted sample was transferred onto prepared agar plates. Sample was allowed to seep into the agar. The process was repeated for each sample dilution. Plates were incubated upside down at 25°C for 120 h. After incubation, we counted colonies on each plate. Calculated average colony count and total yeasts and molds count using formula: TYMC = (average colony count × dilution factor)/Volume of inoculum used. TYMC (colony forming units per gram) was calculated. We, performed positive and negative controls for accuracy. We sterilized equipment, followed good lab practices, and also adhered to safety guidelines.

#### 2.11.3 Enumeration and Isolation of Total Coliform

We prepared 0.1% sterile peptone water or BPW by dissolving 1 g of peptone/BPW in 1 L of sterile distilled water. Violet Red Bile Lactose Agar was prepared according to the manufacturer's instructions and the diluent was autoclaved. Also, 10 g of test sample was weighed and mixed with 90 mL of sterile diluent. We took 1 mL of the mixture, added to 9 mL of sterile water in a dilution tube, and mixed. This was further made to obtain 30–300 colonies on agar plates. Then, 1 mL of diluted sample was transferred to a petri dish. We, added 15–20 mL of molten Violet Red Bile Lactose agar, mixed, and allowed to solidify. Micro‐anaerobic condition was created by adding an over layer. This was then incubated upside down at 35°C or 45°C for 24 ± 2 h. After incubation, we counted colonies on each plate using a colony counter. We selected five colonies, streak on MacConkey Agar, and incubated to confirm lactose fermentation. Total/Fecal coliform counts were calculated, considering confirmed colonies, average colony count, dilution factor, and inoculum volume. We performed positive and negative controls for accuracy. We ensured the equipment was sterilized, followed good lab practices, and also adhered to safety guidelines. This procedure is vital for assessing the microbial contamination levels in a given sample and ensures accurate results through proper dilution, inoculation, incubation, and confirmation steps (Da Silva et al. 2018; Kornacki 2001).

Enumeration of *Staphylococcus Aureus* (Plate Count Method as described in APHA 39.63:2015): Sterile peptone water (0.1%) or BPW were prepared. Sample were weighed, diluted, and mixed thoroughly. Agar plates were prepared with the diluted samples. Diluted sample was transferred to Baird Parker Agar plates. Sample was spread evenly and repeated for different dilutions. Then the plates were incubated and examined for typical *S. aureus* colonies. Colony counts were recorded and we performed coagulase, catalase, and mannitol fermentation tests on selected colonies. *S. aureus* counts were calculated based on confirmed positive colonies. Finally, we used positive and negative controls, sterilized equipment, worked in a sterile environment, and followed safety guidelines.

### 2.12 Statistical Data Analysis

The data were cleaned by checking for inconsistencies, missing values, or outliers; and this data were coded for analysis using statistical software SPSS (version 25). In this study, a total of three therapeutic foods were compared, i.e. TTH and KMC locally formulated RUTF compared to the WHO formulation. Descriptive statistics was calculated for the composition of each therapeutic food sample, including measures such as mean, standard deviation, frequency, and percentages. Thus, samples' differences and acceptability ratings were determined using descriptive analysis and ANOVA. Analysis of variance was employed on the samples means of the 9‐point hedonic scale pertaining to the attributes of acceptability. Statistical significance on the attributes were further analyzed to see where mean differences existed using Post Hoc Test‐LSD at 95% confidence interval. Significant differences or similarities were identified at *p* < 0.05.

## 3 Results

The study involved mothers/caregivers of children from 6 to 59 months old at two hospitals in the Northern region of Ghana. This study largely employed the 9‐point hedonic scale to assess the acceptability of three samples of RUTF (i.e., samples from TTH, KMC, and WHO standard RUTF). Also, the microbial and proximate analyses were performed to determine how safe they were for consumption and whether or not the nutrient values were in line with the WHO guidelines and standards for therapeutic formulation of RUTF for SAM management.

### 3.1 Acceptability and Sensory Evaluation of Samples

The sensory evaluation involved children aged (6–59 months). Table 1 shows acceptability of sensory evaluation of samples. The acceptability ratings provided by mothers and caregivers indicated that, the WHO standard RUTF performed better than the other two samples with an overall acceptability mean of 30.37 followed by TTH 26.91 and KMC was least liked with 22.98. The ANOVA of the samples' differences observed from attributes i.e. appearance, consistency, sweetness, and taste; where all significant (*p* = 0.001). A post Hoc analysis indicates that there was significant difference in all the attributes among the samples except for sweetness and taste between TTH sample and WHO standard formula.

**TABLE 1:** Acceptability and sensory evaluation of RUTF samples (*n* = 112, mothers/caregivers of children 6–59 months).

Attributes	TTH (mean)	KMC (mean)	WHO (mean)	*p*
Appearance	6.30	5.63	8.09	< 0.001
Consistency	5.76	4.64	7.73	< 0.001
Sweetness	7.17	6.04	7.26	< 0.001
Taste/Flavor	7.68	6.67	7.29	< 0.001
Overall	26.91	22.98	30.37	

*Note:* Statistical differences were determined using ANOVA, with *p*‐values reported for comparisons.

### 3.2 Microbial Content of the Local and Standard RUTF

The microbial analysis of the two locally prepared samples from TTH and KMC showed that there was no coliform and *staphylococcus aureus* ( *S. aureus* ) as 0.00 count (CFU/g) was recorded for both hospitals. The microbial analyses values for APC and yeasts for both local therapeutic samples indicate that TTH (APC = 5.50 × 10^1^ ± 0.06, yeasts = 4.00 × 10^1^ ± 1.35) had lower microbial loads compared to KMC (APC = 6.00 × 10^1^ ± 0.00, yeasts = 5.00 × 10^1^ ± 1.41) (Table 2).

**TABLE 2:** Comparison of microbial content of the local and standard RUTF.

Test	TTH count (CFU/g)	KMC count (CFU/g)	m	M	RUTF
APC	5.50 × 10^1^ ± 0.06	6.00 × 10^1^ ± 0.00	1.00 × 10^3^	1.00 × 10^4^	—
Coliform	0.00	0.00	1.00 × 10^1^	1.00 × 10^2^	Negative in 1 g
Yeast	4.00 × 10^1^ ± 1.35	5.00 × 10^1^ ± 1.41	1.00 × 10^2^	1.00 × 10^3^	Maximum 10 in 1 g
*S. aureus*	0.00	0.00	—	—	0 cfu per 25 g

*Source:* Proposed Maximum Toxin level for RUTF adapted from (UNICEF 2023; WHO 2007). Limits; m: distinguishes good quality from marginally acceptable quality. M: distinguishes marginally acceptable quality from defective quality according to Ghana Standard Authority's GS 955:2019 standard for RUTF.

### 3.3 Composition of the Local Formulation Versus Other RUTFs

The nutrient composition analyses showed that with regard to the proteins, TTH recorded 14/100 g, above the WHO RUTF (10%–12%) and KMC with 11/100 g. Energy (kcal/100 g) for KMC was slightly higher (570 kcal) than TTH (530 kcal) and WHO RUTF (520–550); and fats values were 30/100 g for TTH, and KMC sample recorded (40/100 g), which were all lower than WHO RUTF (45%–60%). As far as the mineral analyses were concerned, the local therapeutic samples had seemingly close values to that of the standard, except for the iron content of TTH (19 mg/100 g) and KMC (15.3 mg/100 g) which were higher than the iron levels of 10–14 recorded by the standard RUTF. The magnesium content of TTH (243 mg/100 g) was higher than the KMC (127 mg/100 g), the standard RUTF (80–140) and the other samples compared. The calcium content of TTH (570 mg/100 g) and KMC (330 mg/100 g) were within the range compared to the WHO RUTF, Plumpy nuts, and Nutreal, but the zinc content was generally low for TTH (7.4 mg/100 g) and KMC (5.7 mg/100 g), respectively (Table 3).

**TABLE 3:** Nutrient composition of the local RUTF versus other RUTFs.

Nutrient content	TTH	KMC	WHO RUTF	Plumpy nuts	Nutreal
Protein (g/100 g)	14	11	10%–12%	13.6	15.65
Energy (kcal/100 g)	530	570	520–550	545	545
Carbohydrate (g/100 g)	51	42	—	—	—
Lipids (g/100 g)	30	40	45%–60%	35.7	31.36
Ash (g/100 g)	2.1	0.4	—	—	—
Moisture (g/100 g)	4.2	2.8	2.5*	—	—
Magnesium (mg/100 g)	243 (0.243)	127 (0.13)	80–140	92	98.39
Calcium (mg/100 g)	570 (0.57)	330 (0.33)	300–785	300	512.31
Copper (mg/100 g)	1.3 (0.0013)	0.88 (0.00089)	1.4–1.8	1.8	1.69
Iron (mg/100 g)	19 (0.019)	15.3 (0.0153)	10–14	11.5	12.98
Zinc (mg/100 g)	7.4 (0.0074)	5.7 (0.0057)	11–14	14	13.01
Potassium (mg/100 g)	1660 (1.66)	800 (0.80)	1100–1600	1111	1286.12

*Source:* RUTF figures adapted from (UNICEF 2023; WHO 2007); Plumpy nut figures adapted from (Golden and Grellety 2011).

* Maximum.

### 3.4 Composition of the Local RUTF Versus Liquid Therapeutic Formulae

The nutrient composition analyses indicate that with regard to the proteins TTH (14 g/100 g) and KMC (11 g/100 g) were all higher than F‐75 (0.9 g/100 g) and F‐ 100 (2.9 g/100 g). Energy (kcal/100 g) for KMC (570) and TTH (530) were also higher than F‐75 (75) and F‐ 100 (100); and fats values of 30 g/100 g for TTH, KMC (40 g/100 g) were equally higher than the liquid therapeutic formulae. Pertaining to the mineral values, both F‐75 and F‐100 had lower values than the TTH and KMC minerals composition (Table 4).

**TABLE 4:** Nutrient composition of the local RUTF versus Liquid therapeutic formulae.

Nutrient content	TTH	KMC	F‐75	F‐100
Protein (g/100 g)	14	11	0.9	2.9
Energy (kcal/100 g)	530	570	75	100
Carbohydrate (g/100 g)	51	42	—	—
Lipids (g/100 g)	30	40	—	—
Ash (g/100 g)	2.1	0.4	—	—
Moisture (g/100 g)	4.2	2.8	—	—
Magnesium(mg/100 g)	243 (0.243)	127 (0.13)	0.43	0.73
Calcium (mg/100 g)	570 (0.57)	330 (0.33)	—	—
Copper (mg/100 g)	1.3 (0.0013)	0.88 (0.00089)	0.25	0.25
Iron (mg/100 g)	19 (0.019)	15.3 (0.0153)	—	—
Zinc (mg/100 g)	7.4 (0.0074)	5.7 (0.0057)	2.0	2.3
Potassium (mg/100 g)	1660 (1.66)	800 (0.80)	4.0	6.3

*Source:* RUTF figures adapted from WHO (2007); F‐75, F‐100 values adapted from (UNICEF 2023; WHO 2003); Plumpy nut figures adapted from (Internation 2011).

### 3.5 Composition of the Local Formulations Versus Human Breast Milk

The nutrient composition analyses showed that as far as the proteins were concerned, TTH (14 g/100 g) and KMC (11 g/100 g) recorded higher content in relation to colostrum (2.3 g/100 g) and breast milk (0.9 g/100 g). Energy (kcal/100 g) for KMC (570 kcal) and TTH (530 kcal) were also higher than colostrum (58) and breast milk (58–72); and fats values of 30 g/100 g for TTH and KMC (40 g/100 g) were all high. In terms of the mineral concentrations, both human colostrum and the mature breast milk recorded lower mineral composition compared to samples from the TTH and KMC (Table 5).

**TABLE 5:** Nutrient composition of the local RUTF versus human breast milk.

Nutrient content	TTH	KMC	Colostrum	Breast milk
Protein (g/100 g)	14	11	2.3	0.9
Energy (kcal/100 g)	530	570	58	58–72
Carbohydrate (g/100 g)	51	42	—	—
Lipids (g/100 g)	30	40	2.9	4.2
Ash (g/100 g)	2.1	0.4	—	—
Moisture (g/100 g)	4.2	2.8	—	—
Magnesium(mg/100 g)	243 (0.243)	127 (0.13)	3.4	3.0
Calcium (mg/100 g)	570 (0.57)	330 (0.33)	23	28
Copper (mg/100 g)	1.3 (0.0013)	0.88 (0.00089)	0.25	0.25
Iron (mg/100 g)	19 (0.019)	15.3 (0.0153)	0.045 (45 μg)	0.04 (40 μg)
Zinc (mg/100 g)	7.4 (0.0074)	5.7 (0.0057)	0.54 (540 μg)	0.12 (120 μg)
Potassium (mg/100 g)	1660 (1.66)	800 (0.80)	74	58

*Source:* Colostrum and mature milk adapted data from (Kretchmer and Zimmermann 1997).

### 3.6 Nutrient Content of Local Formulae Versus Dietary Reference Intake of Children

The Recommended Dietary Allowance (RDA) of proteins (1.2 g/kg/day) requirement for children under 5 years was lower than recorded at TTH (14 g/kg/day) and KMC (11 g/kg/day). However, the fat content of the formulae was within the RDA for the age categories. The energy and carbohydrates content of the local samples were lower for the age categories, as (0.5–1) and (1–3) age (years) brackets required 743 kcal/day and 1046 kcal/day of energy, and 95 g/day and 130 g/day of carbohydrates to meet daily requirements. However, TTH and KMC provided 530 kcal/day and 570 kcal/day of energy and 51 g/day and 42 g/day of carbohydrates. Generally, most of the minerals in the local prepared therapeutic samples met the daily requirement, except for the potassium requirements of the under one to 3 years who need as much as 3000 mg/day (Table 6).

**TABLE 6:** Dietary reference intake for children versus the local formulations.

Nutrient content	TTH	KMC	Children (Age in years)	
			0.5–1	1–3
Protein RDA (g/kg/day)	14	11	1.2	1.05
Energy EER (Kcal/day)	530	570	743	1046
Carbohydrate RDA (g/day)	51	42	95	130
Fat AI (g/day)	30	40	30	30–40
Magnesium RDA (mg/day)	243	127	75	80
Calcium AI (mg/day)	570	330	270	500
Copper RDA (mg/day)	1.3	0.88	0.22	0.34
Iron RDA (mg/day)	19	15.3	11	7
Zinc RDA (mg/day)	7.4	5.7	3	3
Potassium AI (mg/day)	1660	800	700	3000

*Source:* Dietary reference intake series adapted from the National Academy Press (Intakes 1997; National Academies of Sciences and Medicine 2019; Trumbo et al. 2001).

Abbreviation: EER, estimated energy requirements.

### 3.7 Participant Feedback on RUTF Products

Thus, in addition to the quantitative acceptability ratings, mothers/caregivers provided their perception pertaining to their experiences with the therapeutic foods. The most notable themes included preferences for taste, texture, and ease of consumption.

### 3.8 Taste Preferences

Many mothers/caregivers indicated their strong opinions about the taste of the therapeutic formulae. A significant number (45 out of 112) reported that the locally prepared formulations were more palatable compared to the standard RUTF. For instance, one respondent indicated that, “My child enjoys the taste of the local RUTF more than the imported one; he finishes it quickly.” Another participant indicated that; “The foreign one is very salty for my child, that is why he keeps spitting it out from his mouth”. However, a considerable number (41 respondents) of the caregivers were of the opinion that, the standard RUTF was good to taste, as expressed by one of the participants that; “It is very good to taste and, very moderate in the sweetness”. These opinions were expressed by a number of the mothers/caregivers, indicating that taste plays a crucial role in the acceptability of therapeutic foods.

### 3.9 Texture and Consistency

The texture of the formulae was another essential factor that influenced acceptability. A considerable number 45 (40%) of the mothers/caregivers opined that, the local formulations were perceived as either too dry or too thick, which made it difficult for some children to consume or swallow it with ease. One participant said, “The RUTF from TTH is very dry; my child struggles to swallow it.” Another participant said, “The RUTF from KMC was rough and gritty, and that is why my child does not like to take it”. This feedback suggests that adjustments in formulation could enhance the sensory experience and improve adherence. However, for the standard RUTF many of the participants 67 (60%) said it was good in texture and that the consistency was just okay, as expressed by one mother that, “I think my child likes this one (i.e the standard RUTF) because it is soft and easy to swallow as compared to the other two foods”.

### 3.10 Cultural Relevance

Participants also highlighted the significance of cultural relevance in the acceptability of RUTF. Many of them mothers 80 (71%) stressed that the recipe used in the local formulations were familiar and hence, aligned with their traditional dietary habits and practices. A mother stated, “I feel good giving my child food that is similar to what we eat at home.” This perception expressed by a number of the participants highlights the potential for locally prepared RUTF to be more acceptable in the various settings, as it resonates with local food preferences.

### 3.11 Ease of Formulation and Use

Mothers/caregivers also commented on the suitability of using locally prepared RUTF. As far as ease of formulation and use of the local RUTF was concerned, 65 participants (58%); appreciated that the local formulations were readily available and easy to prepare. One caregiver mentioned, “I can easily get the ingredients from the market, and it doesn't take long to formulate by the health professionals.” This accessibility may contribute to better adherence to treatment regimens.

### 3.12 Overall Satisfaction

Generally, the feedback indicated a considerable level of satisfaction with the locally prepared RUTF. However, many of participants 50 (45%) expressed their likeness and satisfaction to the foreign standard RUTF based on its performance in the overall evaluation. An appreciable number of caregivers 62 (55%) expressed a willingness to continue using either of the formulations for their children, particularly for the local formulations if improvements in texture and consistency were made. One participant indicated that, “If the texture can be improved, I would prefer this local RUTF for my child, since we know what ingredients are use in it”.

## 4 Discussion

The study evaluated the nutrient composition, microbial content, sensory qualities, and acceptability of locally prepared RUTF from TTH and KMC compared to the WHO standard formulation. The age of participants, from (6 to 59 months), was essential in understanding the sensory results. The feedback from mothers and caregivers highlighted the significance of considering developmental factors when evaluating the acceptability of the different therapeutic foods. Their perceptions provided valuable perspective on how their children responded to the different RUTF formulations, which was essential for tailoring interventions to meet the nutritional needs of young children effectively. The acceptability of the samples in this study indicates that, the WHO samples were well‐liked by participants. Even though the samples were all derived from similar materials, it did not elicit similar responses from the participants. Previous studies looked at locally prepared RUTF compared to other different alternate therapeutic formulae prepared with different ingredients; and the local RUTF performed better in terms of acceptability (Potani et al. 2021; Weber et al. 2017). Again, Weber et al. (2017) indicated locally prepared RUTF performed the same as that of industry manufactured standard RUTF, and thus acceptability and efficacy study by (Thapa et al. 2017), found that locally formulated RUTF (Nutreal) was well‐accepted by the children and not the “defined food” it was compared with. Furthermore, similar studies from other countries have shown locally produced ready‐to‐use food to have better acceptability compared to other types of formulae for managing SAM (Ciliberto et al. 2005; Diop et al. 2003; Isanaka et al. 2009; Manary 2006; Manary et al. 2004; Singh et al. 2010; Yebyo et al. 2013). The mother/caregivers in this study indicated that as far as the likeness to the samples were concerned; the consistency of TTH and KMC formulations were difficult to swallow as they were very dry and not soft for smooth consumption. The integration of their experiences indicates the nuanced observations of the caregivers regarding the RUTF formulations. Thus, the participants expressed a preference for the taste and texture of locally prepared formulations, which is consistent with findings from other studies that emphasize the significance of sensory attributes in the acceptability of therapeutic foods (Potani et al. 2021; Weber et al. (2017)). This underscores the potential for locally formulated RUTF to meet the nutritional needs of children while being culturally appropriate, thereby enhancing adherence to treatment regimens. The UNICEF specification of RUTF formulation indicates that, the paste should not elicit chewing when consumed by the target population and that, sugar particle size, which if not properly ground, could cause oil separation from the paste and lead to oil leakage when opening the sealed part of the sachet (UNICEF 2023). More so, the acceptability ratings from TTH (26.91) and KMC (22.98) could also be attributed to the different skimmed milk products used, as TTH used “Nido Essential” brand and KMC “Lactogen 2”, all products of Nestle‐ Ghana.

The microbial analysis of the two local samples followed the established guidelines for microbial content analysis of RUTF. The safety of the samples from the microbial report revealed that the samples did not contain harmful substance originating from micro‐organisms or any other poisonous substances, including micro‐organisms or foreign matter, in amounts that may present hazard to health. Furthermore, WHO (2007) suggested microbial content analysis of RUTF to include important parameters; coliform, yeast and *S. aureus* . Since already the immune system of SAM children is compromised, the therapeutic formulae are supposed to be formulated under strict hygienic conditions, and safe for consumption.

Relating to the nutrient composition, the protein composition from TTH was slightly higher (14 g) than the WHO RUTF, whereas KMC (11 g) was within the recommended range as compared to the standard RUTF (10%–12%). These disparities could be attributed to differences of nutrient compositions because of differences in the types of materials used for the sample preparations. Also, fats composition of both samples TTH (30%) and (KMC 40%) were lower compared to international guidelines of standard RUTF (45%–60%). The minerals concentrations showed that, potassium from TTH and calcium content from both local samples met the proposed composition for standard RUTF, whiles the rest of the minerals analyzed from the two facilities showed marked disparities of nutrient concentrations as zinc and copper were low and iron was high compared to the WHO standard RUTF. These conspicuous disparities could be due to the added Zincovit since CMV mix was not available. The guidelines stipulate that, quantity of vitamins and minerals added to formulation to achieve the target level must be adjusted based on the chemical form, interaction, and impaired absorption with other nutrients and nonnutrients and scientific evidence showing adequate stability and bioavailability in the finished product (UNICEF 2023). With regard to the iron content between facilities, TTH iron values of 19 mg/100 g was higher than KMC 15.3 mg/100 g, since the milk content used by TTH contained 8 mg/100 g of “Nestle Nido essentia,” compared to 6 mg/100 g of “Nestle Lactogen 2” by KMC. One other very important observation was the high values of moisture content recorded by TTH (4.2 g/100 g) and KMC (2.8 g/100 g) compared to an acceptable maximum threshold of 2.5 g/100 g moisture content by the WHO standard RUTF. The recommendation for RUTF moisture content is indicated to have a maximum level of 2.5 g/100 g to prevent microbial growth (UNICEF 2023; WHO 2007).

The proteins, carbohydrates, fats and the overall energy content of the locally prepared samples had higher values than the liquid F‐75 and F‐100 therapeutic formulae. This indicates that, these local prepared formulae can enhance rapid growth and weight gain for SAM children especially during the rehabilitation phase of SAM management. Also, the potassium, magnesium, zinc, and copper contents of the locally prepared samples per 100 g were also higher compared to the F‐75 and F‐100. This makes the local samples potential formulae alternatives for SAM management and rehabilitation. Better still, when the prepared formulae were compared to the human breast milk; especially proteins, energy content, fats, and carbohydrates; the local formulae performed very well as it had very high nutrient contents compared to the human breast milk. With regard to the micronutrients, the colostrum and breast milk performed low in all the minerals compared, including iron concentration which is reported to be low in human colostrum 0.045 (45 μg) and breast milk 0.04 (40 μg) (Cai et al. 2015; Picciano 2001). It is however, very critical to note that, while breast milk is a critical nutritional source for infants, it was not included in our analysis. Thus, breast milk was not analyzed as part of this study, hence, highlighting the need for future research to explore its role in conjunction with RUTF formulations.

The dietary reference intake for children under 5 years showed, over two‐thirds of the energy requirement for the age (years) groups (0.5–1), and half of requirement of energy for those between 1 and 3 years during nutrition rehabilitation would be met per day by the nutrient contribution of the local samples. Furthermore, the protein content of the samples per 100 g recorded between TTH and KMC were greater than the daily requirements for both infants and children per day. This, indicates how nutrient dense these local formulations are for management of SAM among children under 5 years. The trace minerals content analyzed (copper, zinc and iron) from the two facilities, exceed the RDAs for the children. Also, the calcium and magnesium compared to the RDA for the children were enough to meet the requirements of the children per day. Thus, with the exception of potassium for TTH (1660 mg/day) and KMC (800 mg/day) which were low compared to the (1–3 years) requiring 3000 mg per day, the values for the local samples notwithstanding, had the potential to meet a third of the potassium requirement for that age brackets.

## 5 Conclusion

This study revealed that the nutrient content of the local preparations had potentials to meet the nutrient requirement of children under 5 years, particularly for SAM management. The microbiological examination indicated that the local formulations met the microbial content requirement proposed for RUTF production. TTH and KMC exhibited varying degrees of acceptability, with WHO standard RUTF being preferred overall. The locally prepared RUTF from TTH, showed promise in acceptance, providing a potential sustainable solution for managing SAM. The nutrient analysis of the local RUTF formulations revealed that some formulations contained nutrient levels exceeding the RDAs for certain vitamins and minerals. Therefore, it is essential to consider the implications of excessive nutrient intake, particularly in malnourished children. Hence, careful adjustments are necessary to ensure safety and efficacy, so as to support the growth and recovery of malnourished children while minimizing the risk of adverse effects associated with excessive nutrient intake.

### 5.1 Strengths and Limitations of the Study

The strengths of the study significantly contribute valuable insights to the management of SAM. Thus, the study provided essential information for policymakers, healthcare professionals, and those involved in addressing SAM through a thorough analysis of the nutrient composition, sensory qualities, microbial content, and acceptability of locally formulated RUTF from TTH and KMC. This analysis does not only highlight the potential of locally formulated RUTF but also offers a valuable evaluation to the WHO standard RUTF. Furthermore, this study also emphasizes the potential for improving local preparation methods, which could have an important impact on addressing malnutrition not only in the northern region, but Ghana at large.

However, our study also had its limitations regarding the sample size which was limited to only two hospitals, which may not be representative of the larger population. In addition, the assessment of microbial composition, nutrient content, sensory qualities, and acceptability of the RUTF samples was conducted at a single point in time, without addressing potential changes over time. Furthermore, although we recognized the WHO standard RUTF as generally acceptable, we did not provide a detailed analysis of potential biases in the data. We also acknowledge the limitation of not including sensory evaluations for Plumpy Nut and the other liquid formulas, hence emphasizing the need for future research on a broader range of therapeutic foods to enhance understanding of their effectiveness and user preferences.

## Author Contributions

**Tamimu Yakubu:** conceptualization (supporting), formal analysis (supporting), methodology (supporting), writing – original draft (lead), writing – review and editing (equal). **Charles Apprey:** conceptualization (equal), formal analysis (equal), methodology (equal), writing – review and editing (lead). **Reginald Adjetey Annan:** conceptualization (lead), formal analysis (lead), supervision (lead), writing – review and editing (lead).

## Ethics Statement

This study was approved by the Committee on Human Research, Publication and Ethics at the Kwame Nkrumah University of Science and Technology, Ghana, (CHRPE/AP/730/23).

## Consent

Consent and assent of guardians and participants was obtained before recruitment. The privacy and confidentiality of participants were ensured during data acquisition throughout the study.

## Conflicts of Interest

The authors declare no conflicts of interest.

## Data Availability

The datasets used and/or analyzed during the current study are available from the corresponding author upon reasonable request.
